# Effect of various normalization methods on Applied Biosystems expression array system data

**DOI:** 10.1186/1471-2105-7-533

**Published:** 2006-12-15

**Authors:** Catalin C Barbacioru, Yulei Wang, Roger D Canales, Yongming A Sun, David N Keys, Frances Chan, Karen A Poulter, Raymond R Samaha

**Affiliations:** 1 Molecular Biology Division, Applied Biosystems, Foster City, CA 94404, USA

## Abstract

**Background:**

DNA microarray technology provides a powerful tool for characterizing gene expression on a genome scale. While the technology has been widely used in discovery-based medical and basic biological research, its direct application in clinical practice and regulatory decision-making has been questioned. A few key issues, including the reproducibility, reliability, compatibility and standardization of microarray analysis and results, must be critically addressed before any routine usage of microarrays in clinical laboratory and regulated areas can occur. In this study we investigate some of these issues for the Applied Biosystems Human Genome Survey Microarrays.

**Results:**

We analyzed the gene expression profiles of two samples: brain and universal human reference (UHR), a mixture of RNAs from 10 cancer cell lines, using the Applied Biosystems Human Genome Survey Microarrays. Five technical replicates in three different sites were performed on the same total RNA samples according to manufacturer's standard protocols. Five different methods, quantile, median, scale, VSN and cyclic loess were used to normalize AB microarray data within each site. 1,000 genes spanning a wide dynamic range in gene expression levels were selected for real-time PCR validation. Using the TaqMan^® ^assays data set as the reference set, the performance of the five normalization methods was evaluated focusing on the following criteria: (1) Sensitivity and reproducibility in detection of expression; (2) Fold change correlation with real-time PCR data; (3) Sensitivity and specificity in detection of differential expression; (4) Reproducibility of differentially expressed gene lists.

**Conclusion:**

Our results showed a high level of concordance between these normalization methods. This is true, regardless of whether signal, detection, variation, fold change measurements and reproducibility were interrogated. Furthermore, we used TaqMan^® ^assays as a reference, to generate TPR and FDR plots for the various normalization methods across the assay range. Little impact is observed on the TP and FP rates in detection of differentially expressed genes. Additionally, little effect was observed by the various normalization methods on the statistical approaches analyzed which indicates a certain robustness of the analysis methods currently in use in the field, particularly when used in conjunction with the Applied Biosystems Gene Expression System.

## Background

DNA microarray technology provides a powerful tool for characterizing gene expression on a genome scale. While the technology has been widely used in discovery-based medical and basic biological research, its direct application in clinical practice and regulatory decision-making has been questioned [1,2]. A few key issues, including the reproducibility, reliability, compatibility and standardization of microarray analysis and results, must be critically addressed before any routine usage of microarrays in clinical laboratory and regulated areas can occur. Considerable effort has been dedicated to investigate these important issues, most of which focused on the compatibility across different laboratories and analytical methods, as well as the correlation between different microarray platforms. In this study we investigate some of these issues using the Applied Biosystems Human Genome Survey Microarrays.

The microarrays contain 31,700 60-mer oligonucleotide probes representing 29,098 individual human genes, and uses chemiluminescence (CL) to identify and measure gene expression levels in cells and tissues. In addition to the unique 60-mer probe, an internal control probe (a 24-mer oligonucleotide) is co-spotted with the 60-mer probe on the microarray and labeled with a complementary oligo containing the fluorescent LIZ^® ^dye (FL) during the hybridization of the microarray.

In this study, we analyzed the gene expression profiles of two human tissues: brain and universal human reference sample (UHR). Five technical replicates in three different sites were performed on the same total RNA samples according to manufacturer's standard protocols. Five different methods, quantile [3,4], median [5], scale[6,7], VSN [8] and cyclic loess [6] were used to normalize AB microarray data within each site. Since fold change and variance dependency with intensity is platform dependent [16] we were interested in evaluating the performance of these methods applied to AB microarray data, making this study the first one from this perspective. We restricted our attention on these five methods for the following reasons. These methods are most frequently used normalization methods for AB microarray data. In addition, the microarrays used in this study contain one probe for each gene (for most of the cases), this design restricting the number of normalization methods to be used and making methods based on replicated measurements for each gene (RMA, Plier etc.) inapplicable. Other normalization methods that would also be inapplicable include those explicitly developed for two color technology, or replicated measurements.

1,000 genes spanning a wide dynamic range in gene expression levels were selected for real-time PCR validation. Using the TaqMan^® ^assays data as the reference set, the performance of the five normalization methods was evaluated focusing on the following criteria: (1) Sensitivity and reproducibility in detection of expression; (2) Fold change correlation with real-time PCR data; (3) Sensitivity and specificity in detection of differential expression; (4) Reproducibility of differentially expressed gene lists. The data set analyzed in this manuscript has been reported elsewhere [9] and made publicly available via GEO accession number GSE5350 using the platform GPL 4097 for TaqMan^® ^assays data and GPL 2986 for Applied Biosystems Human Genome Survey Microarrays data.

## Results

### Target selection for real-time PCR validation

**Figure 1:**
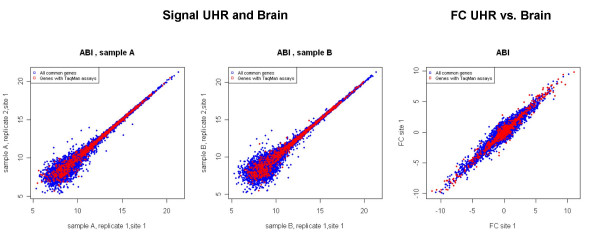
*Gene Selection*: 1000 gene targets were selected for TaqMan^® ^assay validation in order to span a wide dynamic range in expression level and fold changes. Scatter plots between two technical replicates for UHR (A) and Brain (B) samples were shown for the 29,098 genes represented on AB microarrays. The 1000 gene targets are represented in red, and show a wide dynamic range of expression levels and fold change.

In order to conduct a comprehensive survey of the arrays' performance, gene targets for real-time PCR validation were selected based on the following criteria: (1) Ensure a large enough number of validation targets to provide representative overviews of the microarray performance; (2) Select genes spanning a wide range of expression levels and (3) fold changes (Figure 1). 1000 TaqMan^® ^Gene Expression Assays were used in this study, covering 997 genes (3 genes had more than one assay) [[9], MAQC project]. Over 90% of these genes were selected from a subset of 9,442 RefSeq common to the various microarray platforms (Affymetrix, Agilent, GE Healthcare, and Illumina). This selection was designed so that the genes would cover the entire intensity and fold change ranges and include any bias due to RefSeq itself. A subset of (~100) genes were included based on tissue-specificity (UHR versus Brain).

### Sensitivity and reproducibility in detection of expression

**Figure 2:**
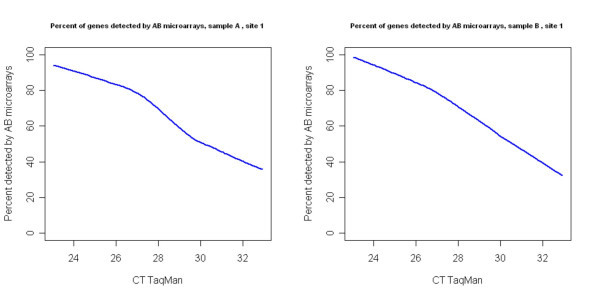
*Detection concordance*: 803 genes for sample A, and 744 genes for sample B are detected as present (CT < 35) for at least two of the TaqMan^® ^assay replicates. For each set of these genes, a sliding window containing 100 consecutive genes was constructed and moved one gene at a time to cover the whole range of Ct values. Within each sliding window, the percent of genes detected as present in at least half of the replicates of individual samples by AB microarray platform was computed and plotted as a function of mean CT value of the 100 genes in the given window.

The dynamic range for the AB microarray platform spans 3–4 orders of magnitude [10], while TaqMan based real-time PCR can achieve 7–8 orders of magnitude dynamic range [11,12]. The larger dynamic range imparts TaqMan^® ^assays with higher detection sensitivity (limit of detection ~1–5 copies per reaction [11]); we therefore used the TaqMan^® ^assays data set as the reference set to evaluate the performance of microarrays in terms of detection sensitivity and accuracy. First, genes that are detectable (positives: above detection threshold) and not detectable (negatives: below detection threshold) were determined for each sample according to manufacturer's recommendations (see Methods for detailed descriptions). Figure 2 shows the relationship between percent genes detected by the microarrays out of the ones detected by TaqMan^® ^assays as a function of CT measurement (number of template transcript molecules is inversely related to C_T_-the more template transcript molecules at the beginning, the lower the C_T_). Gene expression levels were ordered according to TaqMan^® ^assay measurements (average Ct within each sample). A sliding window containing 100 consecutive genes was constructed and moved one gene at a time to cover the whole range of Ct values. Within each sliding window, the percent of genes detected as present in at least half of the replicates of individual samples by the microarray platform was computed out the total of those detected by TaqMan^® ^assays, and plotted as a function of mean CT value of the 100 genes in the given window. The overall sensitivity (True positive Rate (TPR)) and specificity (1-False Positive Rate (FPR)), are presented in Table 1, and are 76.6% and 81.3%, respectively in the UHR sample measured in test site 1.

**Table 1:** Detection concordance between AB microarrays and TaqMan^® ^assays in UHR and Brain.

**UHR, site 1**	**TP**	**TN**	**FP**	**FN**	**Sensitivity**	**Specificity**
**TaqMan**	789	59	0	0	100	100
**AB microarrays**	605	48	11	184	76.68%	81.36%

**Brain, site 1**	**TP**	**TN**	**FP**	**FN**	**Sensitivity**	**Specificity**

**TaqMan**	744	104	0	0	100	100
**AB Microarrays**	581	79	25	163	78.09%	75.96%

For each platform, sample and site, a gene is declared detected (present) if it is detected according to the platform specifications, in more than half of the replicates. Concordance between detection calls are presented in these tables for UHR and Brain samples in site 1 (Sensitivity = true positive rate, Specificity = 1-false positive rate).

**Figure 3:**
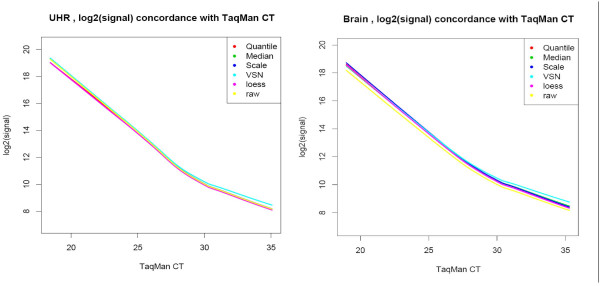
*Signal concordance*: genes detected (present) by TaqMan^® ^assays are used to represent the relationship between expression levels measured by AB microarrays and TaqMan^® ^assays. The average log2(signal) of the 5 replicates from site 1 for all five normalization methods are plotted as functions of gene expression level measured by TaqMan^® ^assays. Lines represent lowess smoothing fitting curves to the set of data points corresponding to one normalization method.

**Figure 4:**
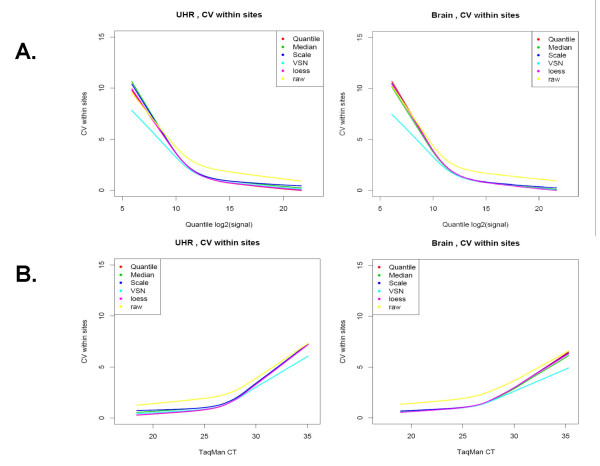
*Reproducibility within sites*: coefficients of variation are used to evaluate the impact of the 5 normalization methods on data reproducibility. (A) presents the CVs, of log2(signal), within site 1 for all 29,098 genes as a function of expression level measured by quantile normalization; (B) presents the CVs within site 1 for genes with TaqMan^® ^assays as a function of TaqMan CT values. Lines represent lowess smoothing fitting curves of all data points from each normalization method.

**Figure 5:**
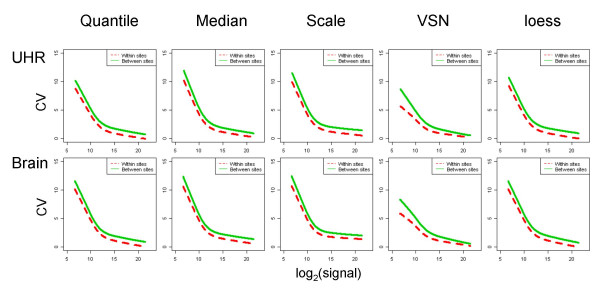
*Variability between sites*: coefficients of variation are used to evaluate the impact of the 5 normalization methods on data reproducibility. One way (site) ANOVA is used to estimate variability within/between sites. CVs within sites (red dotted lines) and between sites (green solid lines) are plotted against quantile normalized data.

For each normalization method, the impact on signal level was determined using genes detected by TaqMan^® ^assays (Figure 3). There are almost no differences in the signal levels when each of these normalization methods is used with the exception of VSN which results in a small increase in signal level for low expressing genes. We also used coefficients of variation (CV) of log_2_(signal) to evaluate the effect of the 5 normalization methods on signal reproducibility, both within and between sites (Figure 4 and 5). Reproducibility of technical replicates for the five normalization approaches for site 1 is illustrated in Figure 4 for both brain and UHR samples. Panel A, where all 29,069 genes are represented, shows the coefficient of variation (CV) across the 5 technical replicates, as a function of expression level when the data is normalized using the quantile approach. Panel B shows the coefficients of variation, only for genes with TaqMan^® ^assays as a function of TaqMan CT. Lines represent the lowess smoothing fitting curves [13] of all data points from each normalization method. As expected, CV's showed a strong dependence on expression level, decreasing from 10% for low expressers or absent genes, to 1% for high expressers. All normalization methods improved the coefficient of variation observed in the raw data over the entire range of expression levels. A small improvement in reproducibility for genes expressed at lower levels was observed in VSN normalization, in both representations, and for both samples. Signal reproducibility between the 3 testing sites is represented in Figure 5. Within sites CVs (dotted lines) and between sites CVs (solid lines) of all 29,098 genes, show similar trends for all five normalization methods. The VSN normalization which showed some improvement in within sites variability of low expression level genes performed similarly to the other normalization methods when between sites variability is considered.

### Fold change concordance with TaqMan^® ^assays

**Figure 6:**
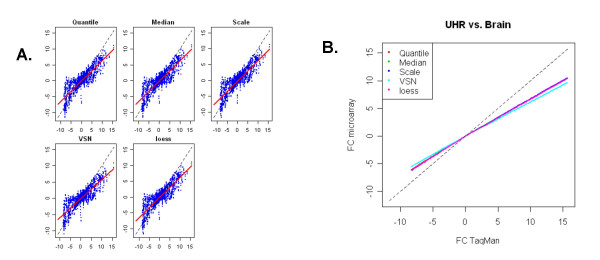
*Fold Change Concordance*: fold change between UHR and brain, determined by each normalization method applied to AB microarray data (y-axis) were plotted against those determined by TaqMan^® ^Assays (x-axis). Genes were filtered based on real-time PCR detection thresholds (detectable in at least 3 out of 4 technical replicates in both samples). (A) linear regression lines (red solid lines) are presented in each plot. (B) lines represent lowess smoothing fitting curves to the 2550 data points (data from all three sites) of each normalization method.

**Figure 7:**
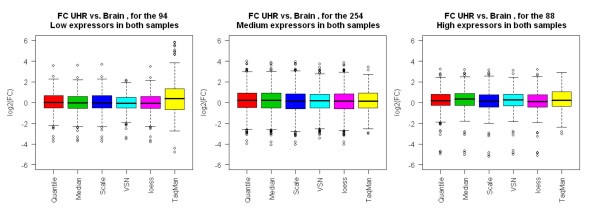
*Fold Change Compression*: Genes were binned into low/medium/high according to TaqMan^® ^assays CT measurements (the cut-offs are set to 24:29:35). Only genes having expression level in the same bin in both sample A and B are included. Boxplots of fold changes for each normalization method and TaqMan assays are presented.

To evaluate the concordance of fold changes between microarray and real-time PCR data, we performed regression analysis of fold differences between the UHR sample (A) and brain sample (B). Fold change metrics are more meaningful as they tend to cancel out systematic platform biases in absolute signal values, moreover they are more biologically relevant. Fold change (log_2_) was computed as the difference in mean expression level of the five technical replicates measured within each site for each sample. Genes were filtered based on real-time PCR detection thresholds (detectable in at least 3 out of 4 technical replicates in both samples) and only genes detected in both samples (848) were used. Fold changes between brain and UHR samples were estimated and plots between (log2) fold changes determined from AB microarray data and TaqMan^® ^assays (ΔΔCt), are presented in Figure 6. The linear regression fitting curve for all data points was performed for each scatter plot (Panel A). The *R*^2^, slope and intercept for TaqMan^® ^assays versus microarray data are presented in Table 2. The 5 normalization methods used for the microarrays showed similar linear correlation characteristics with real-time PCR measurements with R^2 ^values ranging between 0.73–0.75 and slopes ranging from 0.65 to 0.59 suggesting the existence of some ratio compression in the microarray data. VSN showed slightly higher compression than the other normalization methods, most likely caused by the higher signal levels they impart for low expressers (as seen in Figure 3). A similar fold-change comparison between the five normalization methods and TaqMan^® ^assays was also performed using lowess smoothing (Figure 5, Panel B), which does not assume a linear relationship of fold-change values between platforms. The estimated range of fold changes (on log2 scale), for TaqMan assays, is from -8 to 15, while for AB microarrays is -4 to 10. In order to better understand the cause of fold change compression additional analysis was performed on genes with similar expression levels in the two samples. Genes were binned into low/medium/high according to TaqMan^® ^assays CT measurements (the CT cut-offs are set to 23:29:35). Only genes having expression level in the same bin in both samples A and B are included. Boxplots of the fold change for each normalization method and TaqMan^® ^assays are presented in Figure 7. The range of fold changes measured by the microarrays is significantly lower for low expression level genes and somewhat lower for high expression level genes when compared to the range of fold changes measured by TaqMan^® ^assays. For medium expression level genes, the two platforms have a higher agreement on the magnitude of fold differences between the 2 samples.

**Table 2:** *Fold change concordance*: linear regression parameters.

	**Quantile**	**Median**	**Scale**	**VSN**	**Loess**
**Intercept**	-0.095	-0.111	-0.154	-0.127	-0.168
**Slope**	0.639	0.641	0.646	0.589	0.640
**R2**	0.744	0.745	0.744	0.734	0.744

### Sensitivity and specificity in detection of differential expression

**Figure 8:**
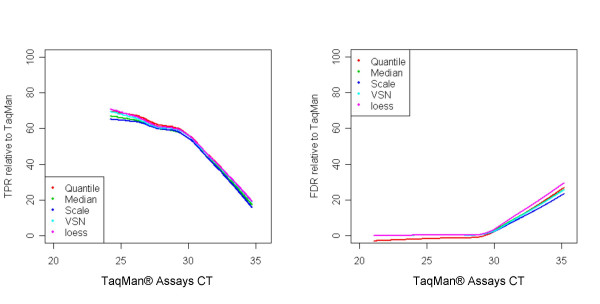
*Significantly differentially expressed genes concordance*: genes detected in both samples by TaqMan^® ^assays were first ranked according to their average CT value in UHR and brain. We use t-test to detect significantly differentially expressed genes, controlling FDR at 5% level. For each bin of 50 consecutive genes (according to the ranking), we compare the results from each normalization method with the ones from TaqMan^® ^assays. We keep track of up/down regulation in each platform. TPR represent the percentage of genes detected differentially expressed in microarray data out of the ones detected by TaqMan assays. FDR was defined as FP/(TP + FP), where FP is false positive in microarray data, and represents the percentage of differentially expressed genes detected only by microarray out of all genes differentially expressed in microarray.

We evaluated the performance of the different normalization methods in detecting differential expression between the two samples using multiple statistical approaches. Traditionally, analysis of accuracy is carried out by analyzing the true positive rate (TPR) and false discovery rate (FDR). In this case, the actual rates are unknown. For this reason, we used TaqMan^® ^as the reference platform. Only genes detected by TaqMan^® ^assays in both samples were used for this section. Using the assay calls as the reference, we constructed contingency tables against microarray data, in which the concordance was determined and both the *P-*value significance of the *t*-test controlling false discovery rate (FDR) at 5% level [14] (i.e. we expect that at most 5% of the genes detected as differentially expressed to be false findings), and fold-change directionality (up- or down regulation) were taken into consideration. Specifically, true positives (TP) are genes differentially expressed (significant *P *value for the *t*-test) in both TaqMan^® ^and microarray platforms with similar direction of the fold change; true negatives (TN) are genes not differentially expressed in both platforms; false positives (FP), consist of two sets of genes: (i) genes not differentially expressed in TaqMan^® ^and differentially expressed in microarrays, or (ii) genes differentially expressed in both platforms with opposite fold change direction; false negatives (FN), genes differentially expressed for TaqMan and not for microarrays. Genes were first ranked according to their average CT value in UHR and brain samples. For each bin of 50 consecutive genes (according to the ranking), we compare the results from each normalization method with the ones obtained with the TaqMan^® ^Assays. TPR defined as TPR = TP/(TP+FN), represents the percentage of genes detected differentially expressed in microarray data out of the ones detected by TaqMan^® ^assays. FDR was defined as FP/(TP + FP), and represents the percentage of differentially expressed genes detected only by the microarrays out of all genes differentially expressed in microarrays. As shown in Figure 8, at the highest expression level, all normalization methods displayed reasonably good sensitivities: 70–75% TPR; the performance drops as the expression level decreases, and at the lowest expression level, the TPR is ~20% (Figure 8, panel A). The opposite trend can be seen for the FDR plots (Figure 8, panel B): a relative constant level of false findings (FDR 1–2%) was observed for genes with high and medium expression levels (CT < 30), after which FDR increases up to 25–30% for genes with low expression level. The overall accuracy of each of the five normalization methods is presented in Table 3. TPR and FDR indicate that the performance of the microarray platform is not dependent on the normalization method used. The observed FDR of 7.1% is slightly higher than the one expected from the FDR control we used to select differentially expressed genes (5%), the 2% overestimate (approximately 28 genes) being possibly explained by genes incorrectly called differentially expressed by the TaqMan^® ^assays (since statistical tests are used to detect differential expression for the TaqMan^® ^Assays, errors are expected to be introduced).

**Table 3:** Significantly differentially expressed genes concordance.

	**TP**	**TP rate**	**TN**	**FP**	**FDR**	**FN**
**TaqMan**	2283	0	261	0	0	0
**Quantile**	1415	61.98	140	101	7.14	888
**Median**	1374	60.18	142	99	7.79	929
**Scale**	1354	59.31	144	97	7.76	949
**VSN**	1393	61.02	142	99	7.69	910
**Loess**	1402	61.41	139	102	7.84	901

Genes detected in both samples by TaqMan^® ^assays (761) are used for this comparison. t-test is used to detect significantly differentially expressed genes, controlling FDR at 5% level. We compare the results from each normalization method, for all sites, with the ones from TaqMan^® ^assays keeping track of up/down regulation in each platform.

**Figure 9:**
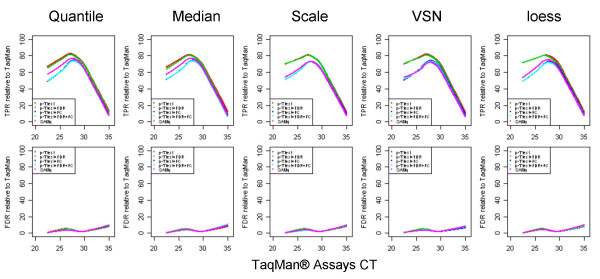
*Differential expression t-test, t-test + FDR, t-test + FC, t-test + FDR + FC cut, SAM applied to Quantile normalization*: we use different methods to detect significantly differentially expressed genes for different normalization methods: (1) t-test (p-value < 0.05), (2) t-test controlling FDR at 5% level, (3) t-test (p-value < 0.05) and FC < 1.5, (4) t-test controlling FDR at 5% level and FC < 1.5, or (5) SAM q < 0.05. We compare the results for data generated by site 1, from each normalization method, with the ones from TaqMan^® ^assays for which differential expression is detected using t-test and controlling FDR at 5% level. We keep track of up/down regulation in each platform.

Additionally, we investigated four commonly used methods for identification of differentially expressed genes in microarray data: simple t-test (p-value < 0.05), t-test combined with fold-change (p-value < 0.05 and FC > 1.5), t-test with FDR and FC control (FDR = 5% and FC < 1.5) and SAM [15] (q-value < 0.05), to determine their impact on the detection of differentially expressed genes. Only data from site 1 was used for this case. Figure 9 shows TPR and FDR plots comparing genes differentially expressed for each statistical method applied to each normalization approach with TaqMan^® ^data used as a reference as previously described (t-test controlling false discovery rate at 5% level). Table 4 presents the overall concordance for Quantile normalized data. The performance of the five methods does not change with the normalization method used. It is important, however, when interpreting these results to take into consideration one particularity of this experiment. The two samples compared, human brain and UHR being extremely divergent tissue types, display big differences in gene expression levels, and so the vast majority of genes used (~90%) showed significant changes (TaqMan^® ^assays). From this perspective, it is expected that FDR control, for genes found differentially expressed by microarrays, will have little impact on FP rates. This explains why the results obtained from the simple t-test and the t-test with FDR control are very similar. In a previous comparative study [16]) where samples with smaller differences were used, we have seen that these two methods show bigger differences in specificity. On the other hand, restrictions on the magnitude of fold change reduce both the number of true positives and false positives. Finally, SAM produces even more specific results (as expected) penalizing some of the low expressers that the TaqMan^® ^assays find differentially expressed.

**Table 4:** Differential expression t-test, t-test + FDR, t-test + FC, t-test + FDR + FC cut, SAM applied to Quantile normalized data.

**Method**	**TP**	**TP rate**	**TN**	**FP**	**FDR**	**FN**
**TaqMan**	763	0	85	0	0	0
**p-t test**	558	73.13	55	30	5.10	205
**p-t test + FDR**	549	71.95	56	29	5.02	214
**p-t test + FC**	483	63.30	64	21	4.17	280
**p-t test +FDR+FC**	479	62.78	64	21	4.20	284
**SAMq**	508	66.58	63	22	4.15	255

We use different methods to detect significantly differentially expressed genes for data Quantile normalized: (1) t-test (p-value < 0.05), (2) t-test controlling FDR at 5% level, (3) t-test (p-value < 0.05) and FC < 1.5, (4) t-test controlling FDR at 5% level and FC < 1.5, or (5) SAM q < 0.05. We compare the results for data generated by site 1, from each normalization method, with the ones from TaqMan^® ^assays for which differential expression is detected using t-test and controlling FDR at 5% level. We keep track of up/down regulation in each platform.

### Reproducibility of differentially expressed gene lists

**Figure 10:**
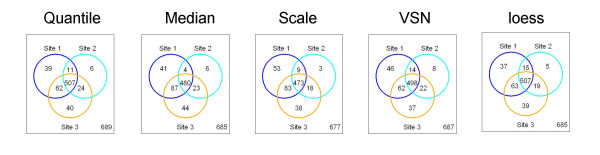
*Reproducibility of differentially expressed gene lists*: we use t-test controlling FDR at 5% level within each site to detect significantly differentially expressed genes for normalized data. We compare the results from each normalization method, across the 3 sites.

A fundamental step in most microarray experiments is determining lists of differentially expressed genes that distinguish biological conditions. Reproducibility of differentially expressed genes across highly similar experiments is one of the important aspects of assessing reliability of microarray results (9, MAQC study). We used Percentage of Overlapping Genes (POG) between differentially expressed genes lists as the measure of reproducibility [9]. For each testing site and each normalization method, we declared genes differentially expressed again using t-test and controlling FDR at 5% level. In this way, for each site we generated a list of differentially expressed genes. Figure 10 shows the overlap between these lists of genes for each normalization method. Table 5 summarizes both percentages and counts of gene overlapping between either pairs of sites or all three sites. One can see that the POG obtained from different normalization methods are similar, ranging from 69.87% (for scale normalized data) to 74.01% (for data loess normalized) when all three sites are compared. Site 2 shows some differences compared to the other two sites, while the comparison between sites 1 and 3 shows consistently 83% POG. Very similar results were observed when the other 4 statistical methods were used for generating gene lists for microarray data (data not shown). It is also important to note that no genes showed discordant results between brain and UHR, i.e. a significant fold change in opposite direction, when different normalization methods were used.

**Table 5:** Reproducibility of differentially expressed gene lists.

**Method**	**Site1&3**	**Site2&3**	**Site1&2**	**Site1&2&3**
**Quantile**	83.3	81.6	79.8	73.58
**Median**	83.5	78.1	75.5	70.07
**Scale**	82.5	78.6	75.4	69.87
**VSN**	82.4	81.7	78.8	72.49
**Loess**	83.8	81.2	80.8	74.01

**Method**	**Site1**	**Site2**	**Site3**	**Common**

**Quantile**	619	548	633	507
**Median**	612	513	634	480
**Scale**	618	501	612	473
**VSN**	620	542	619	507
**Loess**	622	546	628	498

We use t-test controlling FDR at 5% level within each site to detect significantly differentially expressed genes for normalized data. We compare the results for each normalization method, from the 3 sites. First table contains percentage of concordant genes between sites. Second table contains the counts of genes differentially expressed.

## Discussion

One unanswered question in the microarray field has always been the effect of various normalization as well as statistical methods on the end results of a profiling experiment and more explicitly whether using different normalization or statistical approaches results in different gene lists of less concordance between different microarray platforms. In this study we have assessed the performance of five different normalization methods using the Applied Biosystems Expression Array System. Our results show a high level of concordance between these normalization methods. This is true, regardless of whether signals, variation or fold change measurements were interrogated. In addition, these five normalization methods showed similar performance of signal reproducibility between the three testing sites used for this study. Furthermore, we used TaqMan^® ^assays as a reference, to generate TPR and FDR plots for the various normalization methods across the assay range (Figure 8). TPR was directly correlated to gene expression levels whereas FDR was inversely correlated. This is not completely surprising as the two platforms have different dynamic ranges and sensitivity levels, with the detection levels of the microarrays being lower than those of TaqMan^® ^assays. These differences more than likely explain the lower TP rates and higher FP rates for the genes at the low expression levels. These effects were also observed for several other microarray platforms in a separate study [17]. One conclusion of this study is that, at least for the microarray platform tested in this study, the current normalization approaches have little impact on the signal, detection levels as well as TP and FP rates in detection of differentially expressed genes. These results are consistent with the findings of the MAQC study ([18,19]). In addition we also explored the contribution of several statistical approaches commonly used in the field on the TP and FP rates. As expected in this case, with approaches which relax the stringency in differential expression, better detection and differential expression concordance is observed, concomitant with a higher percentage of false positives. At the opposite end of the spectrum, FDR control and SAM methods, which are more restrictive in detection of differential expression, produce gene lists with fewer false positives. SAM, as expected, shows a reduced number of false positives for low expressers, at the expense of missing some differentially expressed genes. The expected percentage of false positives in these lists is close to the one observed when comparing results to TaqMan^® ^assays. Unfortunately, it seems that the full strength of these statistical methods is obscured by the fact that the majority of the genes chosen for TaqMan^® ^validation show significant fold changes between samples, minimizing the effect of FDR on the FP rate. More importantly however, applying the different normalization approaches to the various statistical methods tried, had no significant impact on identifying differentially expressed genes.

Finally, when comparing the overlap in gene lists generated by each of these statistical methods, a concordance of 69.7–74.01% was observed between all three sites, and 82.4–83.8% between sites 1 and 3, indicating little effect of the analysis approach used on the final gene list obtained. This result is, however, sensitive to the cut-offs used in determining the gene lists and can affect the degree of overlap observed [9]. We were pleasantly surprised, however, of the little effect observed by the various normalization on the statistical approaches analyzed which indicates a certain robustness of the analysis methods currently in use in the field.

## Conclusion

In this study we have assessed the performance of five different normalization methods using data generated with the Applied Biosystems Expression Array System. Our results show a high level of concordance between these normalization methods. This is true, regardless of whether signals, variation, site reproducibility or fold change measurements were interrogated. The same similarity is observed when TaqMan^® ^assays were used as a reference, to generate TPR and FDR plots for the various normalization methods across the assay range. In addition we also explored the contribution of several statistical approaches commonly used in the field on the detection of differential expression. Little effect is observed by the various normalization methods on the statistical approaches analyzed which indicates a certain robustness of the analysis methods currently in use in the field, particularly when used in conjunction with the Applied Biosystems microarrays.

## Methods

### RNA samples

#### Sample definition

Sample A was Universal Human Reference RNA (Stratagene) and sample B was human brain total RNA (Ambion).

### Selection of genes for validation by TaqMan assays

A list of 1,297 RefSeqs was selected by the MAQC consortium. Over 90% of these genes were selected from a subset of 9,442 RefSeq common to the four platforms (Affymetrix, Agilent, GE Healthcare and Illumina) used in the MAQC Pilot-I Study (RNA Sample Pilot), based on annotation information provided by manufacturers in August 2005. This selection ensured that the genes would cover the entire intensity and fold-change ranges and include any bias due to RefSeq itself. 1,000 TaqMan gene expression assays were used in the study that matches with the MAQC gene list. These 1,000 assays covered 997 genes (3 genes had more than one assay).

### Applied Biosystems Expression Array analysis

The Applied Biosystems Human Genome Survey Microarray (P/N 4337467) contains 31,700 60-mer oligonucleotide probes representing 29,098 individual human genes. Digoxigenin-UTP labeled cRNA was generated and amplified from 1 μg of total RNA from each sample using Applied Biosystems Chemiluminescent RT-IVT Labeling Kit v 1.0 (P/N 4340472) according to the manufacturer's protocol (P/N 4339629). Array hybridization was performed for 16 hrs at 55°C. Chemiluminescence detection, image acquisition and analysis were performed using Applied Biosystems Chemiluminescence Detection Kit (P/N 4342142) and Applied Biosystems 1700 Chemiluminescent Microarray Analyzer (P/N 4338036) following the manufacturer's protocol (P/N 4339629). Images were auto-gridded and the chemiluminescent signals were quantified, background subtracted, and finally, spot- and spatially-normalized using the Applied Biosystems 1700 Chemiluminescent Microarray Analyzer software v 1.1 (P/N 4336391). Five technical replicates were performed on each sample, at three different testing sites, for a total of 30 microarrays.

### TaqMan^® ^Gene Expression Assay based real-time PCR

#### TaqMan assays

Each TaqMan Gene Expression Assay consists of two sequence-specific PCR primers and a TaqMan assay-FAM™ dye-labeled MGB probe. Each TaqMan assay was run in four replicates for each RNA sample. 10 ng total cDNA (as total input RNA) in a 10:l final volume was used for each replicate assay. Assays were run with 2× Universal PCR Master Mix without UNG (uracil-N-glycosylase) on Applied Biosystems 7900 Fast Real-Time PCR System using universal cycling conditions (10 min at 95°C; 15 sec at 95°C, 1 min 60°C, 40 cycles). The assays and samples were analyzed across a total of 44–384 well plates. Robotic methods (Biomek FX) were used for plate setup and each sample and assay replicate was tracked on a per well, per plate basis.

### Data analysis

Statistical analyses were performed using the open source and open development software project R together with the Bioconductor packages ab1700, limma, multtest and affy [21].

### Normalization methods

When running experiments that involve multiple high density long-oligonucleotide arrays, it is important to remove sources of variation between arrays of non-biological origin. Normalization is a process for reducing this variation. We present five methods of performing normalization at the probe intensity level.

#### Scale normalization

was proposed by Yang et all [6] and is further explained by Smyth and Speed [7]. The idea is to scale the log-ratios to have the same median-absolute-deviation (MAD) across arrays.

#### Global median

The idea is to scale the log-ratios to have the same median across arrays [5].

#### Quantile normalization

Quantile normalization was proposed by Bolstad et al. [3] for Affymetrix-style single-channel arrays and by Yang and Thorne [4] for two-color cDNA arrays. This method ensures that the intensities have the same empirical distribution across arrays.

#### VSN (Variance stabilization normalization)

Based on a function (arsinh) that calibrates for sample-to-sample variations through shifting and scaling, and transforms the intensities to a scale where the variance is approximately independent of the mean intensity [8].

#### Cyclic loess

This approach is based upon the idea of the *M *versus *A *plot, where *M *is the difference in log expression values and *A *is the average of the log expression values, presented in Dudoit *et al*. [20]. However, rather than being applied to two color channels on the same array, as is done in the cDNA case, it is applied to probe intensities from two arrays at a time.

### Signal detection analysis

Detection thresholds are defined according to each platform manufacturer's recommendation. For TaqMan Gene Expression Assays, detection threshold is set as Ct < 35 and Standard deviation (of the 4 technical replicates) < 0.5; for Applied Biosystems Expression Arrays, detection threshold is set as Signal to Noise ratio (S/N) > 3 and quality flag < 5000. Detection in each sample was defined as detectable in 3 out of 4 technical replicates for TaqMan^® ^assays and 3 out of 5 technical replicates within each site for microarrays. Using TaqMan^® ^Gene Expression Assays calls as the reference, contingency tables were constructed against microarrays, in which True Positives Rates (genes detectable by both TaqMan^® ^assay and microarrays as a percentage of all genes detectable by TaqMan assays), are plotted against TaqMan CT values (Figure 1).

### Variability within and between sites for different normalization methods for Applied Biosystems Microarray System

Coefficient of variation (CV) is used to measure variability within each site. In Figure 4 we present the dependency between CV of site 1, with TaqMan CT measurements for each normalization method and each sample. These curves represent the lowess approximation of the CV between the 5 technical replicates of all genes against the CT measurement.

In order to quantify the variability between sites these normalization methods produce, we perform one factor (site) ANOVA on all 29,098 genes. In this way we estimate the percent variability from the total variability (of each gene) that can be explained from site variability (Figure 5). For each gene, CVs are plotted against median expression level measured by quantile normalized data, and lowess fitting curves are used to approximate the all points generated from one normalization method.

### True positive rates and false discovery rates in detection of differentially expressed genes for different normalization methods

In order to have a comprehensive understanding of the performance of these 5 normalization methods, detection of differentially expressed genes between UHR and Brain samples is a key issue. Only genes detected in both samples A and B by TaqMan^® ^assays were used in this comparison. Significantly differentially expressed genes between samples were defined as *p*-value < 0.05 based on a student's t-test controlling FDR at 5% level (BH). Using calls from TaqMan^® ^Gene Expression Assays as the reference, contingency tables were constructed against the different normalization methods, in which we are taking into considerations both *p*-value significance and fold change direction (up or down regulation). Based on this matrix, the TPR, FPR, FDR and accuracy were calculated for each normalization method. Results are presented in Table 3. A more detailed representation of true positive rates and false discovery rates, as functions of CT measurements are presented in Figure 8. Genes were first ranked according to their average value in the tissue comparison. For each bin of 50 consecutive genes (according to the ranking), we compare the results from each normalization method with the ones from TaqMan^® ^Assays. We keep track of up/down regulation in each platform. The average value of these 50 genes in the two samples is plotted against TPR or FDR of the concordance between the two platforms in detecting differentially expressed genes.

## Authors' contributions

CB conceived the study, participated in its design, coordination and performed the statistical analysis and wrote the manuscript. YW participated in the real-time PCR data collection and writing of the manuscript. YS, RC participated in the design of the study, carried out the cross-platform gene mapping and performed the statistical analysis. DK and FC participated in the microarray data collection. KP participated in the real-time PCR data collection. RS contributed to the conception and design of the study, and to revising and writing of the manuscript. All authors read and approved the final manuscript.
